# A Novel Shape Memory Plate Osteosynthesis for Noninvasive Modulation of Fixation Stiffness in a Rabbit Tibia Osteotomy Model

**DOI:** 10.1155/2015/652940

**Published:** 2015-06-08

**Authors:** Christian W. Müller, Ronny Pfeifer, Karen Meier, Sebastian Decker, Janin Reifenrath, Thomas Gösling, Volker Wesling, Christian Krettek, Christof Hurschler, Manuel Krämer

**Affiliations:** ^1^Trauma Department, Hannover Medical School (MHH), Carl-Neuberg-Straße 1, 30625 Hannover, Germany; ^2^Laser Zentrum Hannover e.V. (LZH), Hollerithallee 8, 30419 Hannover, Germany; ^3^Small Animals Clinic, University of Veterinary Medicine, Bischofsholer Damm 15, 30173 Hannover, Germany; ^4^Orthopaedic Department, Hannover Medical School (MHH), Anna-von-Borries-c 1-7, 30625 Hannover, Germany

## Abstract

Nickel-titanium shape memory alloy (NiTi-SMA) implants might allow modulating fracture healing, changing their stiffness through alteration of both elastic modulus and cross-sectional shape by employing the shape memory effect (SME). Hypotheses: a novel NiTi-SMA plate stabilizes tibia osteotomies in rabbits. After noninvasive electromagnetic induction heating the alloy exhibits the SME and the plate changes towards higher stiffness (inverse dynamization) resulting in increased fixation stiffness and equal or better bony healing. In 14 rabbits, 1.0 mm tibia osteotomies were fixed with our experimental plate. Animals were randomised for control or induction heating at three weeks postoperatively. Repetitive X-ray imaging and in vivo measurements of bending stiffness were performed. After sacrifice at 8 weeks, macroscopic evaluation, *µ*CT, and post mortem bending tests of the tibiae were carried out. One death and one early implant dislocation occurred. Following electromagnetic induction heating, radiographic and macroscopic changes of the implant proved successful SME activation. All osteotomies healed. In the treatment group, bending stiffness increased over time. Differences between groups were not significant. In conclusion, we demonstrated successful healing of rabbit tibia osteotomies using our novel NiTi-SMA plate. We demonstrated shape-changing SME in-vivo through transcutaneous electromagnetic induction heating. Thus, future orthopaedic implants could be modified without additional surgery.

## 1. Introduction

Besides biological factors, such as blood supply, cellular immune response, and availability of osteoinductive cytokines, fracture healing strongly depends on adequate biomechanical stimuli [1–3]. While articular fractures require anatomic reduction and heal via direct (primary) bone healing without callus formation, this concept of absolute stability has occasionally led to nonunions, delayed healing, or implant failure in shaft fractures. More mechanically flexible devices for fixation, such as bridging plates or intramedullary nails, have thus been introduced, which allow for indirect (secondary) fracture healing via callus formation [4, 5]. The basic idea behind these more flexible types of fixation is to decrease the strain at the fracture site, which was presumed to be too high after stable fixation of simple fracture types. It is hereby assumed that a larger fracture gap leads to lower tissue strain in the callus for the same magnitude of interfragmentary motion (IFM). It was further postulated that optimal strain would range between the minimum required for the induction of callus and the maximum that allowed bony bridging [4]. Others referred to interfragmentary movements to describe local mechanical conditions needed for adequate bony healing [6, 7].

Physiologically, fracture healing consists of several phases: haematoma formation and inflammation, progression into fibrous tissue, endochondral ossification and formation of hard callus, and remodelling of the bone [8]. It seems likely, that as fracture healing progresses through these phases, the need for stability and IFM changes [7, 9–11]. In simple shaft fractures of femur and tibia, “dynamisation” of osteosynthesis, usually through removal of locking screws of intramedullary interlocked nails six to eight weeks after fracture fixation, is commonly used to lower rigidity of the osteosynthesis and increase loading for the callus [12, 13]. In contrast, Epari et al. recently hypothesized that a more flexible fixation during early stages of fracture healing might result in greater callus formation and that a more rigid fixation at a later stage might promote mineralization of callus, resulting in comparable or maybe faster healing time. This concept was, therefore, called “inverse dynamization” [14]. Glatt et al. named this concept “reverse dynamization,” and applied it in a critical-sized femoral defect model of the rat. In addition to investigating the effect of recombinant bone morphogenetic protein- (BMP-) 2, they used external fixators with different degrees of stiffness and observed an acceleration of bone healing after switching from low to high stiffness two weeks postoperatively [15]. Nonetheless, to our knowledge, no reports of the application of either concept to internal osteosynthesis (plate fixation) have been published to date.

Furthermore, to date, all options of adapting the stability of an internal osteosynthesis in situ have required invasive procedures, such as removing or adding bolts or screws in nail or plate osteosynthesis [16, 17].

Nickel-titanium (NiTi) shape memory alloys (SMA) have the unique ability to return to their original shape after being deformed by warming the material over an alloy-specific temperature [18]. Numerous technical uses for NiTi-SMA have been proposed and implemented [19]. Biomedical applications of SMA include stents for vascular surgery and gastroenterology, wires and brackets in orthodontic surgery, devices for intervertebral body fusion, and staples for use in foot surgery [19]. NiTi-SMA medical devices have shown high mechanical strength and biocompatibility in vitro and in vivo [20–22].

**Figure 1:**
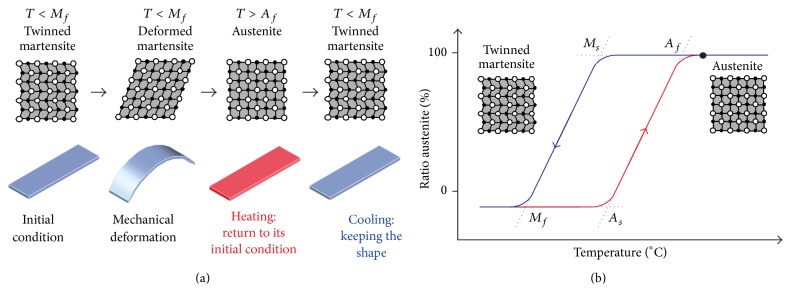
Illustration of the one-way shape memory effect (a) and the hysteresis of martensitic phase transformation (b). *T*: temperature; *A*_*s*(*f*)_: austenite start (finish) temperature; *M*_*s*(*f*)_: martensite start (finish) temperature (principle, actual temperatures depend on the composition of the material). Adapted from [23].

The shape memory effect is based on a reversibly martensitic phase transformation (Figure 1). By cooling the parent phase austenite to a critical temperature *M*_*s*_ (martensite start temperature), the monocrystalline structure of the metal changes into twinned martensite. This transformation ends by reaching the martensite finish temperature (*M*_*f*_). In this state, the martensite can be mechanically deformed, whereby the maximum reversible deformation (*ε*), which can be applied, lies between 6 and 8% (polycrystalline NiTi: 6.7%, nanocrystalline NiTi: 8%) for NiTi-SMAs [24–27]. By raising the temperature above the austenite finish temperature (*A*_*f*_), the martensite converts into austenite again (Figure 1), and the SMA returns to its initial predetermined state, exhibiting the so-called one-way shape memory effect (SME). It should be noted that, in addition to the SME, SMA like other shape change materials can exhibit the so-called shape change or superelasticity or pseudoelasticity effect. This effect is characterized by an instant recovery of shape; however, it occurs at higher temperatures than the SME and is not used in the setup of our study [27, 28].

In other biomedical applications, body temperature is used to convert the implant from the martensite structure to the austenite structure. Contrarily, the composition of the material of our device results in austenite start and finish temperatures significantly above the body temperature, and implant conversion can be triggered by warming the implant at the desired point in time [23, 29]. We have implemented electromagnetic field induction for this purpose. In previous studies, we demonstrated that electromagnetic induction heating can be used to achieve controlled transcutaneous warming of NiTi-SMA specimens located between the quadriceps muscle and the femur or intramedullary in rat models from 40 to 60°C [30, 31]. This is a viable temperature range of austenite finish temperatures (temperatures at which the intended SME is deployed) for our application.

We developed different prototype NiTi-SMA fixation plates, which allowed for in situ variation of bending stiffness mainly due to changes in cross-sectional structure (“dogbone” to straight) by warming the implant above *A*_*f*_. In vitro, we showed increases of up to 160% in equivalent stiffness after warming of a 3 mm thick plate up to 55°C and returning to 37°C in a four-point bending test [21].

**Figure 2:**
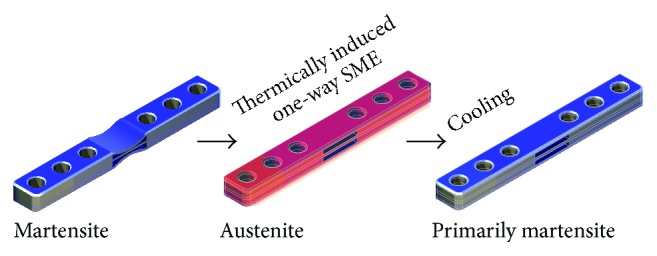
Illustration of the general concept of change of configuration of the experimental plate.

The present study was designed to test the hypothesis that a novel NiTi-SMA plate stabilizes tibia osteotomies in rabbits and allows for bony healing. After noninvasive electromagnetic induction heating the alloy exhibits the SME and the plate changes its configuration towards higher stiffness resulting in increased fixation stiffness of the osteosynthesis (Figure 2).

In addition, we aimed to evaluate whether this “inverse dynamization” affects efficacy of bony healing compared to fixation with the same plate without altering its stiffness.

## 2. Materials and Methods

### 2.1. Animal Model

All operations and procedures were approved by the state veterinary administration (TV 10/170) and complied with the Animal Protection Act of Germany. Adult male New Zealand rabbits (*n* = 14, weight 2.90–3.02 kg) were operated after randomization for subsequent noninvasive induction heating of the implant or a control group without heat induction. Animals were caged according to the guidelines of the European Commission (ETS 123, 2003) and fed with 100 g of pelletized special rabbit food and a small piece of apple per day. Postoperative analgesia (Carprofen) and antibiotics (Baytril) were subcutaneously administered once per day for 10 days. Postoperatively, animals were visited daily and checked for signs of pain or infection.

### 2.2. SMA Implants

A six-hole plate was designed using CAD-software (SolidEdge, Siemens PLM Software, Germany). Dimensions were chosen to match those of a regular 2.0 mm AO small animal plate (DCP) for the use in small dogs [32]. Plates were constructed using a nickel-titanium shape memory alloy with a nominal composition of 49.8 to 50.0% Ni (rest: Ti) and an intermediate transformation temperature (*A*_*f*_ ~ 55°C, Memry GmbH, Weil am Rhein, Germany). NiTi-SMA sheets measuring 0.5 and 1.0 mm in thickness were used in a sandwich construction. Segments were cut using a cutting laser FLS 352 N (Rofin-Lasag AG, Thun, Switzerland) and subsequently welded together using a pulsed Nd:YAG laser system True Pulse 103 (Trumpf, Ditzingen, Germany). After mechanical bevelling of the screw holes, the implants were cleaned using an ultrasonic bath.

The plates were autoclaved at 121°C for 90 minutes prior to implantation and mechanically deformed to the “dog shape” configuration using a standard needle holder (Figure 1).

### 2.3. Induction Device

The induction heating process was performed using a water-cooled generator-oscillator combination as described before (HFG 10, Eldec Schwenk Induction, Dornstetten, Germany) [30, 31]. The induction coil was made of three copper windings with an inner diameter of 150 mm leaving enough room to place a rabbit's hind leg inside the coil. As before, a frequency of 250 kHz was used to minimize the impact of the electromagnetic field on biological tissue [33, 34].

A control loop was integrated within the induction system for the purpose of increasing the temperature of the NiTi-SMA plates to a specified temperature. In order to determine suitable induction parameters, first warming experiments were performed in vitro [29]. The induction system was then validated in a series of preliminary experiments on a rabbit cadaver. In these experiments, the local temperature was measured using fibre-optic probes placed adjacent to the plate (probe TS 5; evaluation unit FoTemp 1; OPTOcon, Dresden, Germany). Due to both the small heat dissipation and the small heat capacity, this method offers an accurate temperature measurement (accuracy ± 1°C, resolution 0.1°C).

### 2.4. Operative Procedure

All procedures were performed under general anaesthesia. Premedication was intramuscularly applied using 5 mg midazolam, 25 mg ketamine, 0.15 mg buprenorphine, and 0.1 mg glycopyrronium bromide. General anaesthesia was induced using propofol 1 mg/kg body weight and maintained with 2% isoflurane and oxygen at a flow rate of 1500 mL/min after orotracheal intubation. Baytril 2.5% 7.5 mg/kg was administered subcutaneously for prophylaxis of infection. The animal was placed in a supine position. The right hind limb was shaved, cleaned, disinfected, and draped. A 5 cm skin incision was performed and the anteromedial aspect of the tibia was exposed. The plate was provisionally fixed and holes for all screws were drilled using a 1.6 mm drill. An osteotomy was performed in the middle of the inner holes using an oscillating saw, resulting in a 1.0 mm gap. The plate was fixed with bicortical 2.0 mm screws (Synthes, Umkirch, Germany). In addition, four 1.6 mm Kirschner wires were drilled parallel to the plate position into the tibia with two on each side of the osteotomy in order to allow for later in vivo measurements of stiffness. The wound was irrigated and closed with absorbable sutures. Kirschner wires were shortened and a sterile dressing and a soft cast were put on.

### 2.5. Radiographic Examination

Under general anaesthesia, conventional X-ray images in two planes were obtained immediately after surgery and again after 3, 4, 5, and 6 weeks. X-ray images were reviewed for signs of implant dislocation, fractures, and bone healing.

### 2.6. In-Vivo Stiffness Measurement

A validated 4-point-bending stiffness device was used as previously described [35]. Under general anaesthesia, rabbits were placed supine on the test rig. External fixators were mounted using the two proximal pins and the two distal pins, respectively. Load was applied on the two inner pins using different weights. The deflection of the system was measured by an eddy current system that was temporarily fixed to the pins on one side of the model. Deflection measurements were repeated ten times per load and means were calculated. As described before, loads were increased from 0 g (0 N) in increments of 25 g (0.25 N) up to 300 g (3.0 N) and further in increments of 50 g (0.5 N) up to a maximum of 700 g (7.0 N) with a resulting maximum moment of 81.42 ± 3.65 Nmm [35]. Deflection was monitored; at a maximum of 40 *μ*m the measurements were stopped to avoid damage of the callus [36]. Structural bending stiffness was calculated as Nm^2^ according to ISO9585:1990(E) and ASTM F382-99 after measurement of the exact position of the K-wires relative to the osteotomy in X-rays standardised for magnification factor (see Supplementary Figure 1 in Supplementary Material available online at http://dx.doi.org/10.1155/2015/652940).

### 2.7. Induction Heating

Following the in vivo stiffness measurement three weeks after the procedure, in animals randomized for induction heating, the right hind limb was placed in the induction device. Based on prior in vivo and in vitro experiments, the initial induction heating parameters were set to 5 seconds at a machine power of 3 kW and thereby an estimated power uptake of 40 ± 5 W [21, 29–31]. Then, X-ray images were carried out to check for change of configuration of the plate. As configuration change was not evident after induction heating as described, further induction heating was performed under the control of a sterile temperature probe placed adjacent to the plate using a peripheral venous catheter. Additional induction heating was performed until X-ray controls revealed a straight configuration.

Subsequently, postinduction in vivo stiffness measurement was done.

### 2.8. Sample Preparation

After euthanasia of the animals six weeks postoperatively, the right and left tibiae were harvested and dissected from all soft tissue. The bone plates were removed and all bones were covered with a bandage moistened with 0.9% saline. Six native tibiae (contralateral side, no procedure) were randomly chosen to serve as a control group.

### 2.9. Micro-CT (*μ*CT)

Tibiae were preserved in synthetic cylinders that fit into the *μ*CT scanner. The osteotomy regions of all specimens were then imaged with a *μ*CT scanner (*μ*CT 80, Scanco Medical AG, Brüttisellen, Switzerland) at 15–90 *μ*m spatial resolutions. Three-dimensional images were calculated and the newly built bone within the osteotomy gap was analysed. The bone volumes as well as trabecular thicknesses were calculated on a Hewlett Packard AlphaStation DS25 computer (Scanco Medical *μ*CT Tomography, Brüttiselen, Switzerland).

### 2.10. Ex-Vivo Bending Structural Stiffness Tests

The specimens then underwent a series of nondestructive four-point bending tests according to the protocol described in ISO9585:1990(E) and ASTM F382-99 using a universal materials-testing machine (Mini Bionix 858, MTS Systems, Minneapolis, USA, Supplementary Figure 2). All tests were carried out in coronal plane with a maximum moment of 330 Nmm at a speed of 2 mm/min. Each test was repeated a total of five times. The linear portion of the load-deformation curve was used to calculate the structural bending stiffness of the bones. Five loading cycles were performed; the first two cycles served as preconditioning of the samples and the subsequent three cycles were used for calculating average stiffness.

### 2.11. Statistical Analysis

The level of significance was set at *p* < 0.05. The null hypothesis was that no differences would be present between the groups (induction and control). Mann-Whitney *U* test was used to analyse *μ*CT data and results from in vivo stiffness measurement. For statistical analysis of the ex vivo bending test the Kruskal-Wallis test was used to determine the similarity of the medians of the three groups.

## 3. Results

### 3.1. Complications

Two animals (one per group) suffered fractures distant to the osteotomy gap during implantation of the plates, which could be successfully managed with additional lag screw fixation. No other intraoperative complications occurred. One animal of the control group broke its hind leg two days postoperatively after getting stuck between two bars of the cage and was sacrificed early. There were no signs of undue pain and no signs of infection in any of the remaining animals postoperatively. One animal bit off parts of the dressing and the suture of the skin; after irrigation and wound closure, the wound healed primarily and healing was otherwise uneventful. One rabbit of the induction group died during transport to the X-ray unit after the induction heating procedure. Therefore, six animals per group remained until the end of the experiment.

### 3.2. Macroscopic Evaluation

**Figure 3:**
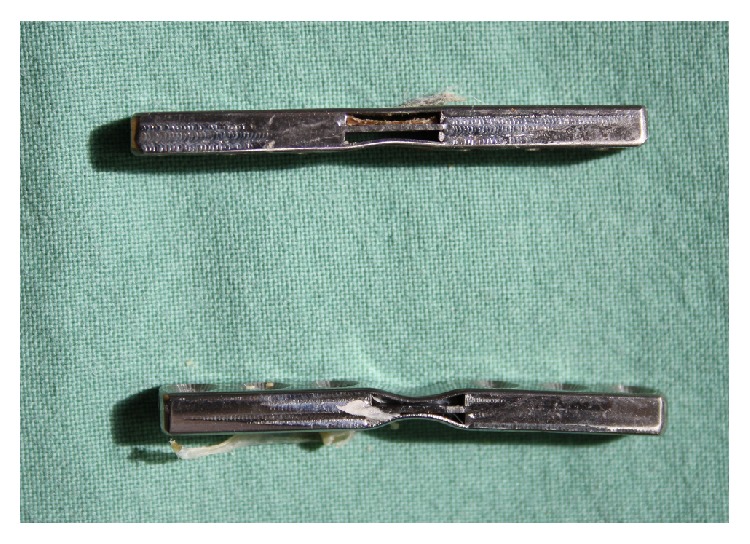
Typical configuration of the plates at sacrifice (transformed plate from the stiffening group, top; untransformed plate from the control group, bottom; plates from animal 30 and 85).

After sacrifice, macroscopically primary healing of the osteotomy was apparent in all remaining 12 cases. No dislocations of implants occurred. In all animals from the induction group, in the middle section of the plate the outer NiTi-SMA layer (averted from the bone) was straight, while the part facing the bone displayed only partial straightening (Figure 3). In animals from the control group, the plate configuration was maintained from the time of implantation (dogbone).

### 3.3. Radiographic Evaluation

**Figure 4:**
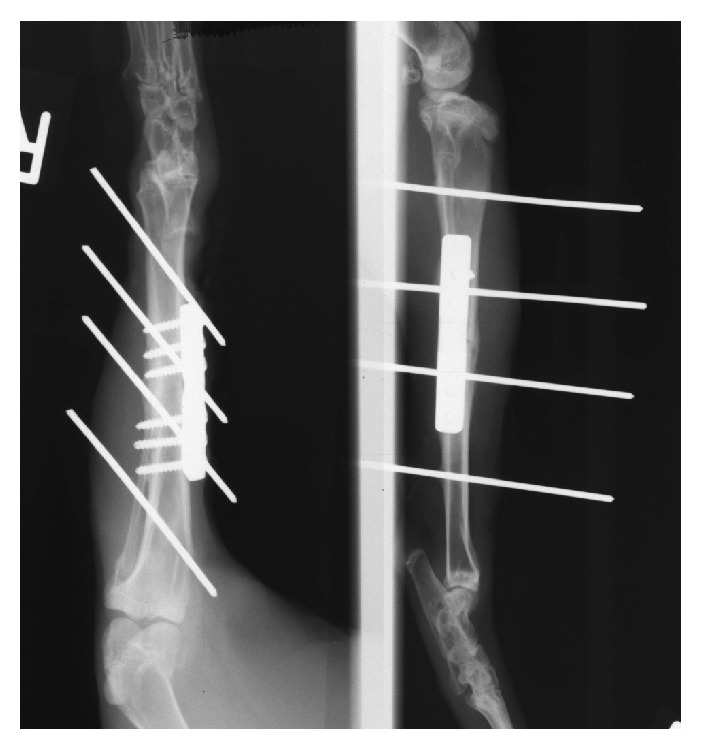
Biplanar radiographs of the right tibia at 42 d postoperatively (animal 30) show conversion of the plate to straight configuration as well as callus formation at the osteotomy site.

There were no signs of implant loosening or displacement. At three weeks, callus formation and partial osseous healing (bridging of the osteotomy gap) were evident in all cases. At 6 weeks, osseous healing of the fracture was apparent in all but one case. Final X-ray images in two planes after transcutaneous induction heating showed successful transformation from dogbone to straight configuration of the plate in all cases (cf.  Figure 4).

### 3.4. In Vivo Stiffness Measurement

In three animals (all of them control group), the position of the Kirschner wires prevented application of the in vivo measurement device. Full sets of measurements were, therefore, available in nine subjects (six of them induction group). In the induction group, the average structural bending stiffness increased from 0.19 ± 0.15 Nm^2^ at 16 ± 1 days to 0.76 ± 0.27 Nm^2^ at 58 ± 1 days postoperatively (*p* = 0.043), whereas in the control group, the average bending stiffness increased from 0.10 ± 0.11 Nm^2^ at 19 ± 4 days to 0.67 ± 0.79 Nm^2^ at 55 ± 3 days postoperatively (n.s.). Differences between both groups were not significant. Due to a technical breakdown of the measurement device, in three animals of the treatment group, the postinduction measurement was carried out one week after the induction procedure. Comparison between structural bending stiffness just before induction heating and the first measurement after revealed a mean increase of bending stiffness of the tibiae from 0.50 ± 0.44 Nm^2^ at 22 ± 4 days to 0.60 ± 0.31 Nm^2^ at 24 ± 3 days postoperatively (n.s.).

### 3.5. Ex Vivo Bending Tests

**Figure 5:**
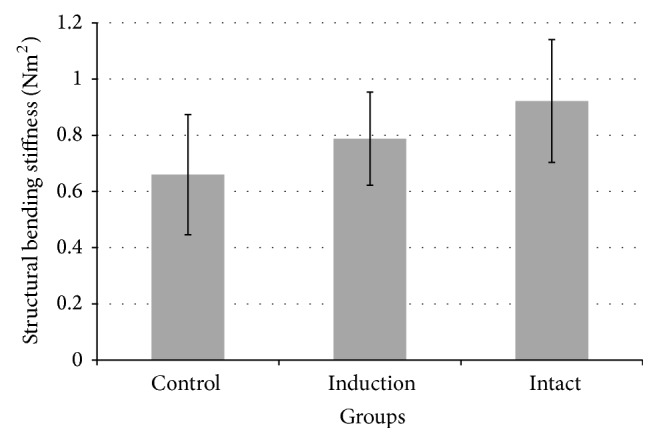
Structural bending stiffness of right tibiae after explantation of the plates (“induction” and “control”) and left tibiae of same animals (“intact”).

The control osteotomy group without induction showed a mean structural bending stiffness that was 72% (0.66 ± 0.21 Nm^2^) of the “intact” (contralateral) group (0.92 ± 0.22 Nm^2^). The induction group showed a mean bending structural stiffness of 85% (0.79 ± 0.17 Nm^2^) relative to the intact group (Figure 5). No significant differences in the bending structural stiffness between the intact, induction, and control groups were observed (*p* = 0.11).

### 3.6. *μ*CT

After the loss of two animals as described above, six specimens per group were available for investigation. Mean bone volumes per total volumes were 55.7 ± 3.0% in the induction group, and 49.2 ± 3.2% in the control group (n.s.). Mean trabecular thickness was 0.119 ± 0.017 mm and 0.095 ± 0.010 mm, respectively (n.s.). Supplementary Figure 3 shows an example of a 3D reconstruction of the scanned area of a tibia from the induction group. Detailed information as to the *μ*CT analysis is presented in Supplementary Table 1. Differences between same parameters in both groups were not significant.

## 4. Discussion

In this study, a novel NiTi shape memory alloy plate was used to stabilize tibiae in rabbits after osteotomy. At the time of sacrifice, bony healing was apparent macroscopically and radiologically. Induction heating in the treatment group resulted in deployment of the SME and change of configuration of the plate towards higher stiffness, shown radiologically and macroscopically after sacrifice. In vivo stiffness measurements showed high intra- and interindividual variations. Differences between groups were not significant. Bending tests following sacrifice demonstrated a trend for higher structural bending stiffness of the tibiae in the induction group relative to the control group. Differences for the volume of newly built bone at the osteotomy gap were not significant in the *μ*CT analysis.

To our knowledge this is the first study of its kind, reporting data on the use of a new concept of internal fixation in long bones, which allows increasing fixation stiffness during the course of healing without additional surgery. As with our earlier experiments, in which small NiTi-SMA specimens beneath rats' femora were used, contactless electromagnetic induction heating proved to be efficient in raising the temperature of a much bigger implant above the austenite finish temperature [31]. Furthermore, our results confirm our previous in vitro data on the change of bending stiffness equivalent of our NiTi-SMA plate after inducing the one-way SME [21].

In the past, various attempts have been made to influence bone healing by optimizing biomechanical parameters [1, 37–45]. However, to date, these attempts were restricted either to refining fixation at the time of surgery, secondary surgery, or the use of external fixation. The only way to achieve a change of mechanical properties of an internal osteosynthesis without additional surgery so far has been the use of degradable materials, for example, polylactide, which has been used for anterior fixation of fusion procedures in the cervical and lumbar spine. Yet, this concept may not be transferable for fracture treatment of long bones. Furthermore, the course of degradation is not controllable, and absorption has been shown to lead to inflammatory reactions [46–50].

Several limitations of this study should be considered when drawing conclusions. As this is the first in vivo application of this new technique, we were not able to calculate the number of animals needed for an adequate sample size estimate. In this study, we used an osteotomy model to represent long-bone fracture in a small animal model. Certain inherent differences exist between fracture and osteotomy situations with respect to soft-tissue damage, cytokine release, and bony involvement. However, it has been shown that bone healing after osteotomy very closely resembles regular fracture healing [51].

Although animals were randomized for treatment or control group, the workflow did not allow blinding of the researcher in regard to the in vivo measurement. Nor was there a feasible way to do a sham induction heating, as the effect was clearly visible in X-ray controls. In vivo stiffness measurements as performed in this study reflect the stiffness of the combination of plate, bone, and K-wires rather than the stiffness of the plate alone. However, it seems unlikely that either bone or K-wires change their stiffness between the measurements before and after the induction process, and measuring the stiffness of the whole system might even better reflect the strain distribution at the fracture site which is always a combination of fracture pattern, fixation, and load. The exact position of the K-wires relative to the position of the plate might influence the absolute measurement of stiffness; therefore, only intraindividual changes and not absolute values of stiffness can be compared [1, 35].

Due to its limited use so far, NiTi-SMA as an implant material might raise concerns with regards to biocompatibility. However, earlier studies have not shown significant adverse effects in experimental orthopaedic applications [52, 53]. Furthermore, although depending on the composition of the material, the temperature needed to achieve the shape memory effect could be as low as 40 to 45°C, our own previous studies did not reveal significant local or systemic inflammatory reactions after induction heating of extra- and intramedullary NiTi-SMA implants in rats at induced temperatures of up to 60°C [30, 31]. Nonetheless, induction heating could still be painful if used without adequate analgesia. Electromagnetic induction heating had to be performed longer than expected from prior data; therefore, it seems advisable to perform the induction procedure under control of an image intensifier. From a technical point of view, in vivo measurements using the apparatus as shown above are technically demanding and time-consuming. General anaesthesia is required in order to avoid movements of the animal, which could prevent reproducible measurements. As the stiffness of the bone-plate construct depends on the direction in which load is applied, the position of the Kirschner wires, which are used to fix the apparatus to the bone, is crucial for the achievement of reliable results.

## 5. Conclusion

To the best of our knowledge, this study reflects the first attempt to apply the idea of noninvasive inverse dynamization to an internal osteosynthesis in vivo. A novel NiTi shape memory plate was used for stabilization of an experimental tibia fracture in rabbits. Noncontact electromagnetic induction heating of the plate three weeks after the initial procedure resulted in a change of its shape towards a state of higher stiffness. Fracture healing was unimpaired and successful in all cases. However, we were unable to measure changes of in vivo implant stiffness between pre- and postinduction. Neither could we show significant differences in bending stiffness of the tibiae ex vivo or in bony structure by *μ*CT between both groups.

Nonetheless, the ability of applying the shape memory effect noninvasively might allow for adaptation of the implant depending on the individual course of fracture healing. Therefore, our findings encourage the further development of novel concepts for long-bone fracture fixation with variable stiffness, which might be used in the future to improve fracture healing and prevent or reduce nonunion.

## Supplementary Material

Supplementary Figure 1: Calculation of structural bending stiffness from in-vivo stiffness measurements.
Supplementary Figure 2: Setup of the ex-vivo bending-test.
Supplementary Figure 3: μCT 3D reconstruction of the osteotomy region of the tibia below the explanted plate (animal 30, induction group) shows bony bridging of the osteotomy 42 days after surgery.
Supplementary Table 1: Data from μCT analysis of rabbit tibiae after explantation.
