# Supplementary material for: A Novel Shape Memory Plate Osteosynthesis for Noninvasive Modulation of Fixation Stiffness in a Rabbit Tibia Osteotomy Model
Source: Biomed Res Int. 2015 Jun 8;2015:652940. doi: 10.1155/2015/652940 (PMC4475735; doi:10.1155/2015/652940)
Supplement: Supplementary file 1 — Supplementary Figure 1: Calculation of structural bending stiffness from in-vivo stiffness measurements. Supplementary Figure 2: Setup of the ex-vivo bending-test. Supplementary Figure 3: μCT 3D reconstruction of the osteotomy region of the tibia below the explanted plate (animal 30, induction group) shows bony bridging of the osteotomy 42 days after surgery. Supplementary Table 1: Data from μCT analysis of rabbit tibiae after explantation. [file 652940.f1.pdf]

Müller CW et al.,

A novel shape memory plate osteosynthesis for non-invasive modulation of fixation stiffness in a rabbit tibia osteotomy model

Supplementary material

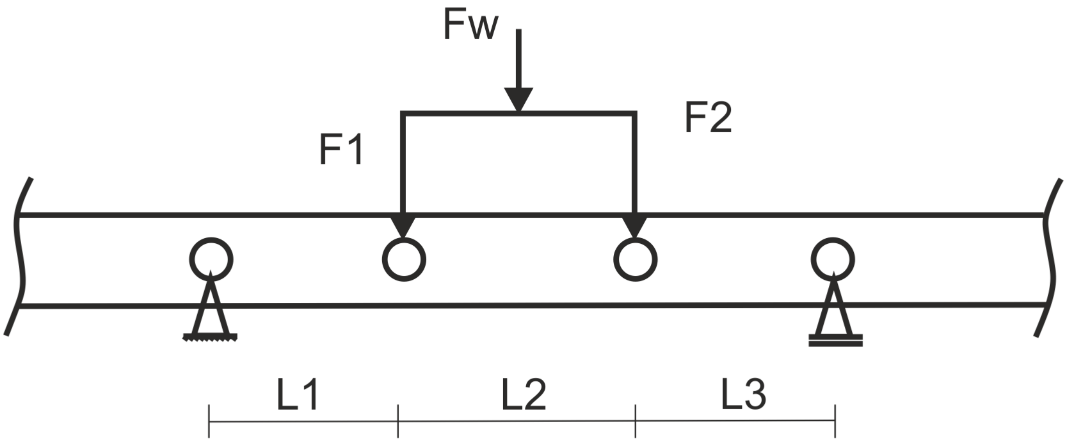

Supplementary figure 1: Schematic illustration of the in vivo four-point bending test. Showing weight (F_w), distance between proximal pins (L_1), distance between inner pins (L_2), distance between distal pins (L_3). Calculation of structural bending stiffness from in-vivo stiffness measurements

with $\frac{\Delta F_w}{\Delta w} = m\ (Slope\ of\ the\ Force - Distance - Graph)$ and $L_{total} = L_1 + L_2 + L_3$

$$EI = m * \frac{L_{total}^3}{6} * \left[\left(\frac{L_1}{L_{total}} \right)^2 * \left(\frac{L_2 + L_3}{L_{total}} \right)^2 + \left(\frac{L_1 + L_2}{L_{total}} \right)^2 * \left(\frac{L_3}{L_{total}} \right)^2 \right]$$

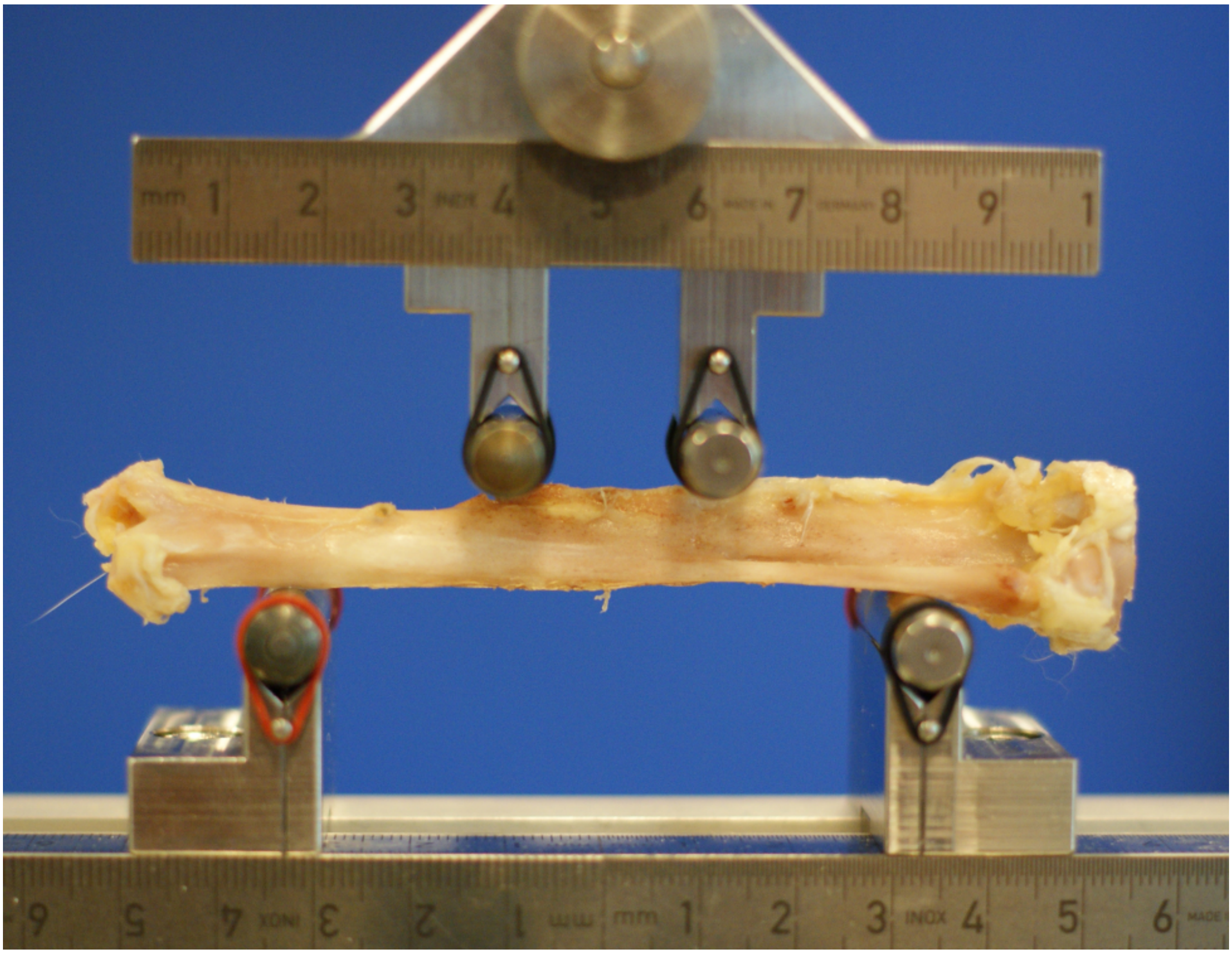

Supplementary figure 2: Setup ex-vivo four-point bending-test

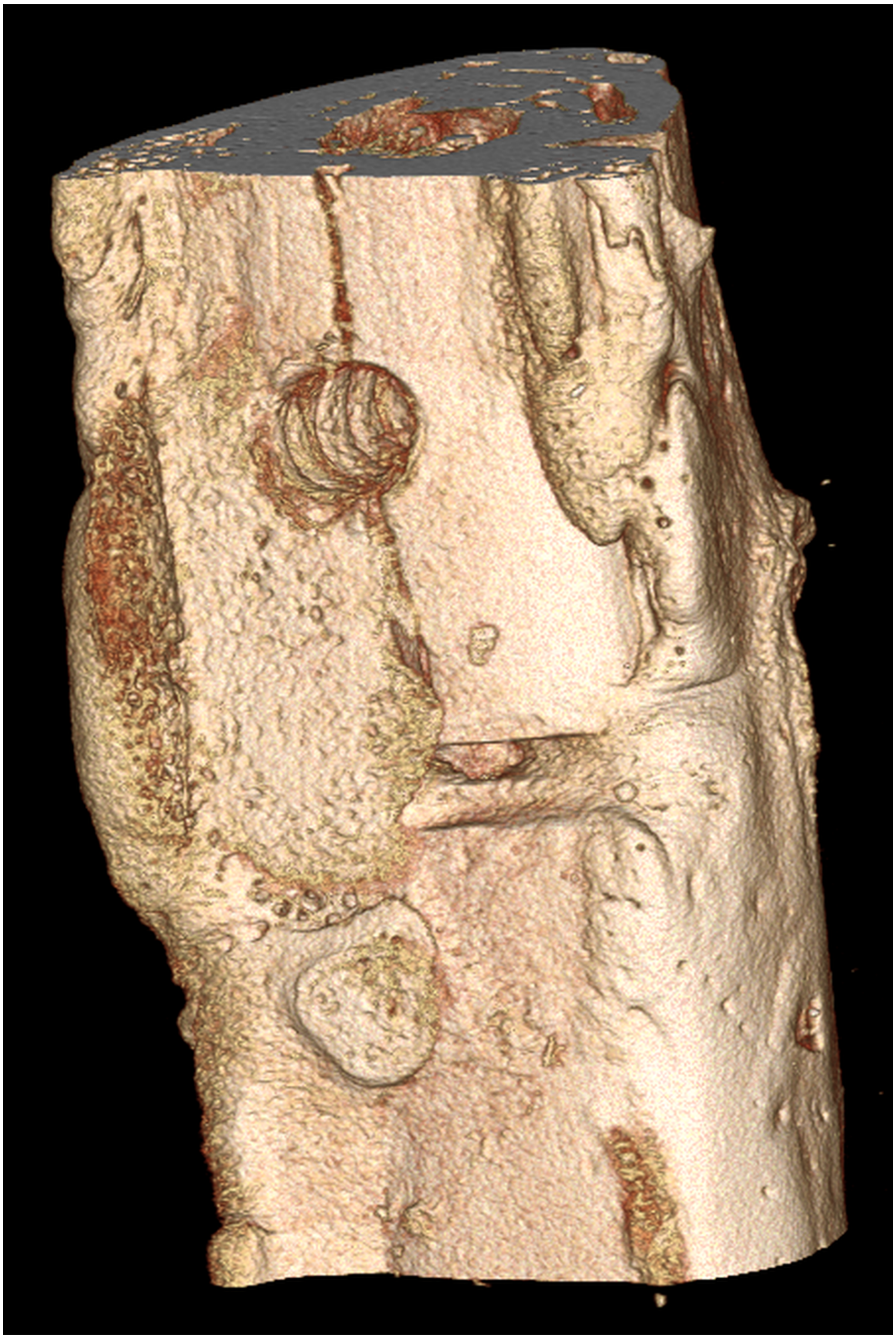

Supplementary figure 3: µCT 3D reconstruction of the osteotomy region of the tibia below the explanted plate (animal 30, induction group) shows bony bridging of the osteotomy 42 days after surgery.
